# Supplementary material for: Longitudinal relations between non-suicidal self-injury and both depression and anxiety among senior high school adolescents: a cross-lagged panel network analysis
Source: PeerJ. 2024 Oct 7;12:e18134. doi: 10.7717/peerj.18134 (PMC11466236; doi:10.7717/peerj.18134)
Supplement: Supplemental Information 3 [file peerj-12-18134-s003.pdf]

supplemental materials

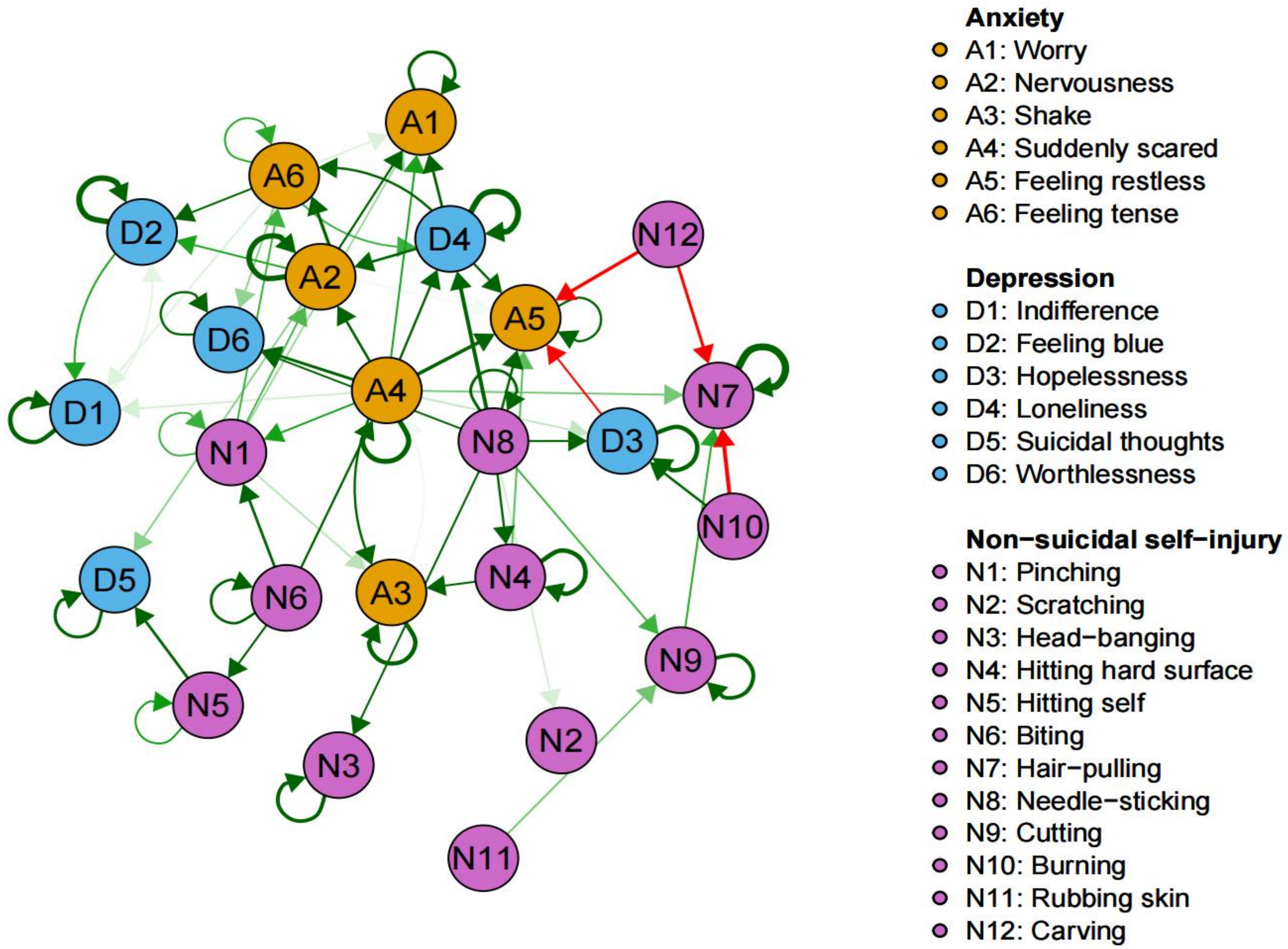

Fig. S1. The cross-lagged panel networks (including all cross-lagged edges) in total sample. Green edges indicate positive relationships, and red edges indicate negative relationships. The arrows between symptoms reflect cross-lagged associations, where symptom a → symptom b indicates that symptom a at time 1 predicts symptom b at time 2, controlling for all other associations and covariates.

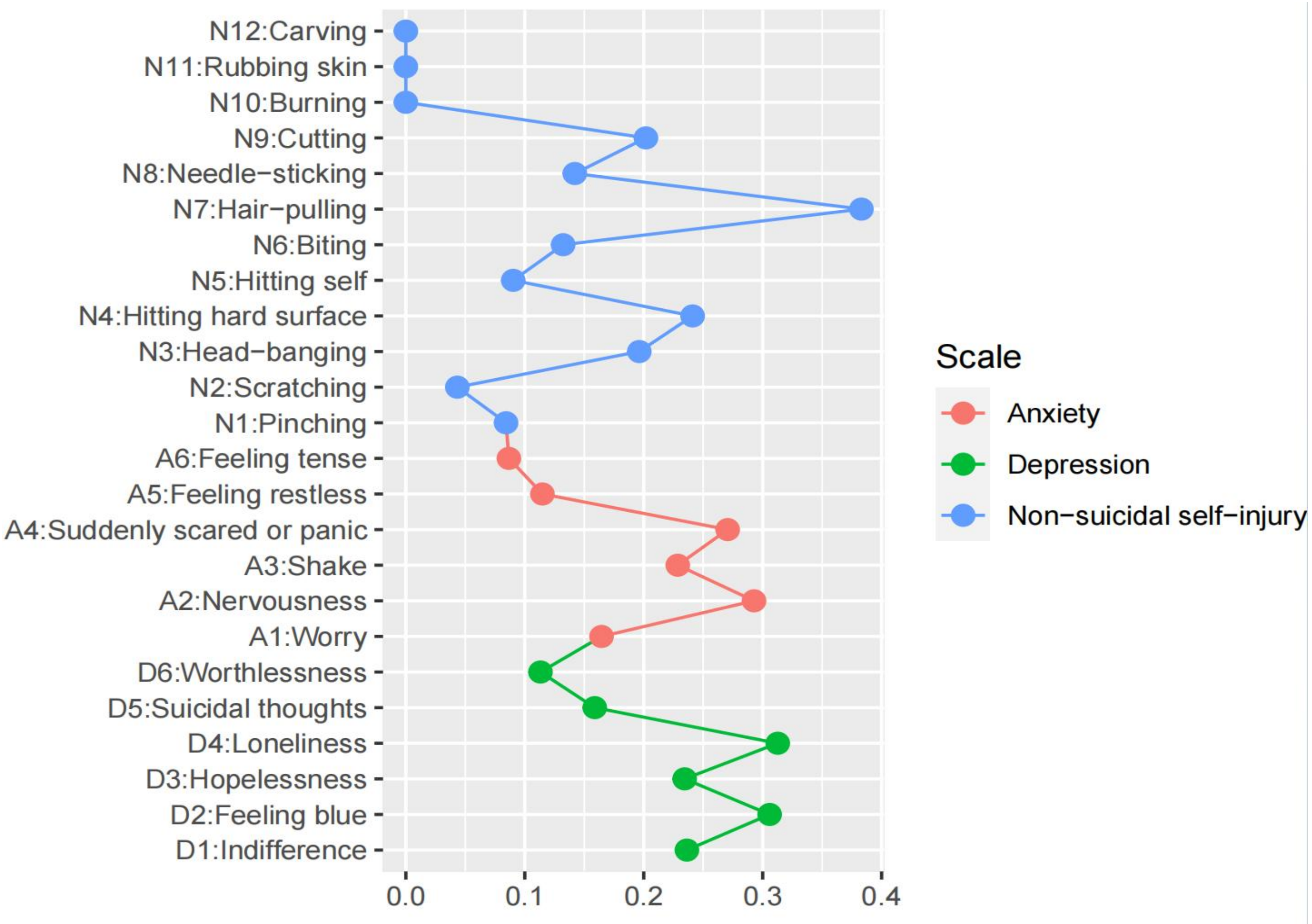

Fig. S2. Autoregressive edges for each symptom in total sample.

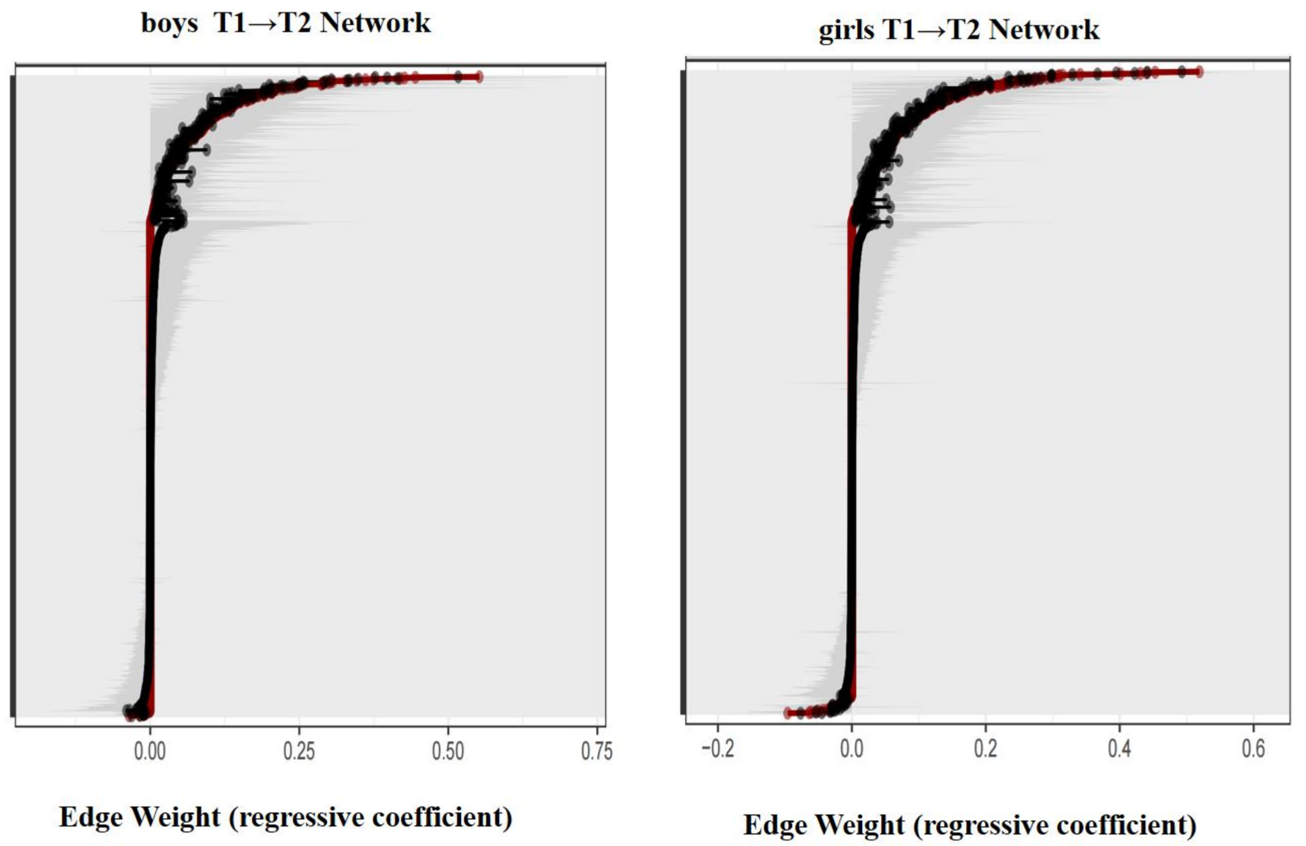

Fig. *S3*. Bootstrapped 95% confidence intervals around each edge weight.

Table S1 Regularized regression coefficients for each cross-lagged network association in total sample

	T2D1	T2D2	T2D3	T2D4	T2D5	T2D6	T2A1	T2A2	T2A3	T2A4	T2A5	T2A6	T2N1	T2N2	T2N3	T2N4	T2N5	T2N6	T2N7	T2N8	T2N9	T2N10	T2N11	T2N12
T1D1	0.24	0.06	0.00	0.00	0.00	0.00	0.00	0.00	0.00	0.00	0.00	0.00	0.00	0.00	0.00	0.00	0.00	0.00	0.00	0.00	0.00	0.00	0.00	0.00
T1D2	0.09	0.31	0.00	0.00	0.00	0.00	0.01	0.01	0.01	0.00	0.06	0.03	0.00	0.01	0.00	0.00	0.00	0.00	-0.01	0.00	0.00	0.00	0.00	0.00
T1D3	0.00	0.00	0.23	0.00	0.00	0.00	0.00	-0.05	0.00	0.00	-0.10	0.00	0.02	0.05	0.00	0.00	0.00	0.00	0.00	0.06	0.05	0.00	0.00	0.00
T1D4	0.00	0.00	0.03	0.31	0.02	0.04	0.13	0.13	0.05	0.06	0.12	0.12	0.00	0.00	0.00	0.00	0.00	0.00	0.00	0.00	0.00	0.00	0.00	0.00
T1D5	0.05	0.04	0.02	0.00	0.16	0.00	0.00	0.03	0.00	0.00	0.00	0.00	0.00	0.00	0.00	0.00	0.00	0.00	0.01	0.00	0.00	0.00	0.00	0.00
T1D6	0.03	0.00	0.00	0.00	0.00	0.11	0.00	0.00	0.00	0.00	0.00	0.00	0.00	0.00	0.00	0.00	0.00	0.00	-0.02	0.00	0.00	0.00	0.00	0.00
T1A1	0.00	0.00	0.00	0.00	0.00	0.05	0.16	0.00	0.00	0.00	0.00	0.03	0.00	0.00	0.00	0.00	0.00	0.00	0.00	0.00	0.00	0.00	0.00	0.00
T1A2	0.06	0.09	0.05	0.03	0.07	0.03	0.11	0.29	0.03	0.06	0.04	0.16	0.00	0.00	0.00	0.00	0.00	0.00	0.01	0.01	0.00	0.00	0.00	0.00
T1A3	0.00	0.00	0.00	0.00	0.03	0.00	0.00	0.01	0.23	0.06	0.06	0.01	0.00	0.00	0.00	0.04	0.00	0.00	0.00	0.00	0.00	0.00	0.00	0.00
T1A4	0.07	0.04	0.07	0.11	0.00	0.15	0.09	0.12	0.11	0.27	0.18	0.05	0.09	0.05	0.00	0.00	0.00	0.01	0.08	0.00	0.00	0.00	0.00	0.00
T1A5	0.00	0.00	0.00	0.00	0.02	0.01	0.01	0.02	0.00	0.00	0.11	0.00	0.00	0.00	0.00	0.00	0.00	0.00	0.00	0.00	0.00	0.00	0.00	0.00
T1A6	0.06	0.09	0.00	0.08	0.04	0.07	0.06	0.06	0.02	0.01	0.05	0.09	0.00	0.00	0.00	0.00	0.00	0.00	0.00	0.00	0.00	0.00	0.00	0.00
T1N1	0.00	0.01	0.00	0.02	0.00	0.06	0.07	0.08	0.07	0.05	0.01	0.08	0.08	0.00	0.00	0.00	0.00	0.00	0.00	0.00	0.00	0.00	0.00	0.00
T1N2	0.00	0.00	0.01	0.00	0.00	0.00	0.00	0.00	0.00	0.00	0.00	0.00	0.00	0.04	0.00	0.00	0.03	0.00	0.00	0.00	0.00	0.00	0.00	0.00
T1N3	0.06	0.00	0.00	0.00	0.00	0.00	0.00	0.00	0.00	0.00	-0.04	0.00	0.00	0.00	0.20	0.00	0.00	0.00	0.00	0.00	0.00	0.00	0.00	0.00
T1N4	0.00	0.02	0.00	0.00	0.03	0.00	0.05	0.04	0.10	0.04	0.08	0.04	0.00	0.00	0.00	0.24	0.00	0.02	0.00	0.00	0.00	0.00	0.00	0.00
T1N5	0.00	0.00	0.00	0.00	0.14	0.05	0.00	0.03	0.05	0.00	0.04	0.01	0.00	0.00	0.00	0.05	0.09	0.00	0.04	0.00	0.00	0.00	0.00	0.00
T1N6	0.04	0.00	0.00	0.00	0.03	0.00	0.00	0.00	0.00	0.11	0.06	0.00	0.14	0.03	0.03	0.01	0.10	0.13	0.00	0.00	0.00	0.00	0.00	0.00
T1N7	0.00	0.00	0.00	0.00	0.00	0.01	0.00	0.00	0.00	0.00	0.05	0.00	0.00	0.00	0.00	0.04	0.00	0.00	0.38	0.00	0.00	0.00	0.00	0.00
T1N8	0.00	0.00	0.10	0.20	0.00	0.09	0.04	0.00	0.00	0.00	0.12	0.04	0.02	0.07	0.10	0.11	0.01	0.00	0.00	0.14	0.08	0.00	0.00	0.06
T1N9	0.00	0.00	0.00	0.00	0.00	0.00	0.00	0.00	0.00	0.00	0.00	0.00	0.00	0.00	0.00	0.00	0.00	0.00	0.09	0.00	0.20	0.00	0.00	0.04
T1N10	0.00	0.00	0.12	0.00	0.00	0.00	0.00	0.02	0.00	0.00	0.00	0.00	0.00	-0.04	0.00	0.00	0.00	-0.06	-0.20	-0.02	0.00	0.00	0.00	0.00
T1N11	0.00	0.00	0.00	0.00	0.00	0.00	0.00	0.00	0.00	0.00	0.00	0.00	0.00	0.00	0.00	0.00	0.00	0.00	0.00	0.00	0.08	0.00	0.00	0.00
T1N12	0.00	0.00	0.00	0.00	0.00	0.00	0.00	0.00	0.00	0.00	-0.18	0.00	0.00	0.00	0.00	0.00	0.00	-0.01	-0.15	0.00	0.00	0.00	0.00	0.00

Note. Adjacency matrix of the T1 → T2 cross-lagged panel network in total. Independent variables (i.e., predictors) are in columns, and dependent variables are in rows. Autoregressive edges are presented along the diagonal. A = anxiety; D = depression; N = non-suicidal self-injury.

Table S2 Regularized regression coefficients for each cross-lagged network association in boys

	T2D1	T2D2	T2D3	T2D4	T2D5	T2D6	T2A1	T2A2	T2A3	T2A4	T2A5	T2A6	T2N1	T2N2	T2N3	T2N4	T2N5	T2N6	T2N7	T2N8	T2N9	T2N10	T2N11	T2N12
T1D1	0.30	0.20	0.01	0.00	0.00	0.00	0.01	0.00	0.00	0.00	0.00	0.00	0.00	0.00	0.02	0.00	0.00	0.00	0.00	0.00	0.00	0.00	0.00	0.00
T1D2	0.08	0.28	0.00	0.00	0.00	0.01	0.07	0.01	0.00	0.01	0.08	0.07	0.00	0.00	0.02	0.00	0.00	0.00	0.00	0.00	0.00	0.00	0.00	0.00
T1D3	-0.04	-0.31	0.00	0.00	-0.04	0.00	-0.23	-0.26	-0.09	-0.09	-0.26	-0.16	0.00	0.00	-0.11	0.00	0.00	0.00	0.00	0.00	0.00	0.00	0.00	0.00
T1D4	0.00	-0.06	0.00	0.13	0.00	0.00	0.09	0.00	0.04	0.00	0.07	0.00	0.00	0.00	-0.05	0.00	0.00	0.00	0.00	0.00	0.00	0.00	0.00	0.00
T1D5	0.00	0.12	0.02	0.00	0.09	0.00	-0.02	0.00	-0.01	-0.01	-0.02	0.00	0.00	0.00	0.00	0.00	0.00	0.00	0.00	0.00	0.00	0.00	0.00	0.00
T1D6	0.00	-0.09	0.00	0.00	0.00	0.04	0.00	0.01	0.00	0.00	0.00	0.00	0.00	0.00	-0.02	0.00	0.00	0.00	0.00	0.00	0.00	0.00	0.00	0.00
T1A1	0.00	0.00	0.00	0.00	0.00	0.00	0.17	0.00	0.00	0.00	0.00	0.00	0.00	0.00	0.00	0.00	0.00	0.00	0.00	0.00	0.00	0.00	0.00	0.00
T1A2	0.00	0.01	0.04	0.02	0.00	0.01	0.07	0.18	0.00	0.05	0.02	0.12	0.00	0.00	0.00	0.00	0.00	0.00	0.00	0.00	0.00	0.00	0.00	0.00
T1A3	0.00	0.00	0.00	0.00	0.08	0.00	0.00	0.00	0.20	0.06	0.00	0.00	0.00	0.00	0.00	0.00	0.00	0.00	0.00	0.00	0.00	0.00	0.00	0.00
T1A4	0.01	0.03	0.05	0.05	0.00	0.14	0.18	0.27	0.14	0.30	0.28	0.13	0.01	0.00	0.09	0.00	0.00	0.00	0.03	0.00	0.00	0.00	0.00	0.00
T1A5	0.00	0.01	0.00	0.00	0.04	0.00	0.00	0.08	0.02	0.00	0.10	0.02	0.00	0.00	-0.02	0.00	0.00	0.00	0.03	0.00	0.00	0.00	0.00	0.00
T1A6	0.10	0.23	0.00	0.11	0.12	0.12	0.04	0.05	0.07	0.00	0.05	0.14	0.00	0.00	0.03	0.00	0.00	0.00	0.00	0.00	0.00	0.00	0.00	0.00
T1N1	0.05	0.42	0.00	0.03	0.00	0.00	0.00	0.35	0.00	0.00	0.10	0.06	0.15	0.00	0.00	0.00	0.00	0.00	0.00	0.05	0.00	0.00	0.00	0.00
T1N2	0.00	-0.48	0.00	0.00	0.00	0.00	0.00	-0.30	0.00	0.00	0.00	0.00	0.00	0.00	0.03	0.00	0.00	0.00	0.00	0.00	0.00	0.00	0.00	0.00
T1N3	0.03	0.19	0.00	0.00	-0.01	0.00	0.00	0.00	0.00	0.00	-0.32	0.00	0.00	0.00	0.25	0.00	0.00	0.00	0.00	0.00	0.00	0.00	0.00	0.00
T1N4	0.04	0.14	0.00	0.00	0.05	0.08	0.12	0.07	0.13	0.06	0.10	0.05	0.00	0.00	0.02	0.27	0.00	0.00	0.00	0.00	0.00	0.00	0.00	0.00
T1N5	0.33	0.30	0.01	0.00	0.22	0.00	0.16	0.32	0.18	0.18	0.38	0.27	0.00	0.00	0.03	0.00	0.00	0.00	0.00	0.00	0.00	0.00	0.00	0.00
T1N6	0.14	-0.06	0.00	0.00	0.00	0.00	0.05	-0.08	0.00	0.00	0.00	0.00	0.00	0.00	-0.35	0.00	0.00	0.00	0.00	0.00	0.00	0.00	0.00	0.00
T1N7	0.08	0.04	0.07	0.02	0.01	0.06	0.16	0.17	0.08	0.00	0.09	0.08	0.00	0.00	0.02	0.00	0.00	0.00	0.34	0.00	0.00	0.00	0.00	0.00
T1N8	0.15	0.01	0.00	0.00	0.00	0.00	0.26	0.00	0.00	0.16	0.14	0.00	0.00	0.00	0.46	0.00	0.00	0.05	0.00	0.00	0.00	0.00	0.00	0.00
T1N9	-0.49	-0.57	0.00	0.00	-0.31	0.00	-0.29	-0.46	-0.09	-0.34	-0.16	-0.52	0.00	0.00	-0.23	0.00	0.00	0.00	0.00	0.00	0.00	0.00	0.00	0.00
T1N10	-0.07	0.17	0.04	0.00	-0.05	0.00	-0.29	0.17	-0.16	-0.09	-0.21	0.06	0.00	0.00	-0.02	0.00	0.00	0.00	0.00	0.00	0.00	0.00	0.00	0.00
T1N11	-0.47	-0.18	0.00	0.00	-0.16	0.00	0.00	-0.08	0.00	-0.02	-0.19	-0.05	0.00	0.00	-0.34	0.00	0.00	0.00	0.00	0.00	0.00	0.00	0.00	0.00
T1N12	0.00	-0.16	0.00	0.00	0.00	0.00	-0.14	-0.05	0.00	0.00	-0.23	-0.19	0.39	0.00	0.48	0.00	0.00	0.00	0.00	0.00	0.00	0.00	0.00	0.00

Note. Adjacency matrix of the T1 → T2 cross-lagged panel network in boys. Independent variables (i.e., predictors) are in columns, and dependent variables are in rows. Autoregressive edges are presented along the diagonal. A = anxiety; D = depression; N = non-suicidal self-injury.

Table S3 Regularized regression coefficients for each cross-lagged network association in girls

	T2D1	T2D2	T2D3	T2D4	T2D5	T2D6	T2A1	T2A2	T2A3	T2A4	T2A5	T2A6	T2N1	T2N2	T2N3	T2N4	T2N5	T2N6	T2N7	T2N8	T2N9	T2N10	T2N11	T2N12
T1D1	0.23	0.02	0.00	0.00	0.00	0.00	0.00	0.00	0.00	0.00	0.00	0.00	0.00	0.00	0.00	0.00	0.00	0.00	-0.01	0.00	0.00	0.00	-0.01	0.00
T1D2	0.08	0.31	0.00	0.00	0.00	0.00	0.00	0.00	0.01	0.00	0.01	0.00	0.00	0.03	0.00	0.00	0.00	0.00	0.00	0.01	0.00	0.00	0.00	0.00
T1D3	-0.03	0.00	0.35	0.06	0.00	0.00	0.00	0.00	0.00	0.00	0.00	0.00	0.02	0.05	0.00	0.00	0.01	0.00	0.00	0.10	0.12	0.00	0.00	0.00
T1D4	-0.03	0.00	0.01	0.36	0.03	0.01	0.11	0.14	0.04	0.09	0.10	0.18	0.00	0.00	0.00	0.01	0.00	0.01	0.00	0.00	0.00	0.00	0.00	0.00
T1D5	0.13	0.08	0.03	0.00	0.24	0.00	0.04	0.09	0.10	0.08	0.06	0.05	0.00	0.00	0.00	0.02	0.00	0.00	0.00	-0.01	0.00	0.00	0.00	0.00
T1D6	0.17	0.09	0.05	0.00	0.00	0.17	0.04	0.00	0.00	0.00	0.00	0.03	0.00	0.00	0.00	0.00	0.00	0.00	-0.02	0.00	0.00	0.00	0.00	0.00
T1A1	-0.05	0.01	0.00	0.00	0.00	0.05	0.12	0.00	0.00	0.00	0.00	0.04	0.00	0.00	0.00	0.01	0.00	0.00	0.00	0.00	0.00	0.00	0.00	0.00
T1A2	0.11	0.13	0.01	0.01	0.13	0.02	0.11	0.34	0.09	0.04	0.06	0.15	0.00	0.00	0.00	0.00	0.00	0.00	0.00	0.00	0.00	0.00	0.00	0.00
T1A3	0.00	0.03	0.02	0.00	0.00	0.04	0.00	0.05	0.23	0.05	0.09	0.05	0.00	-0.02	0.00	0.02	0.00	0.01	0.00	0.00	0.00	0.00	0.00	0.00
T1A4	0.16	0.07	0.08	0.14	0.00	0.14	0.06	0.03	0.08	0.25	0.11	0.01	0.09	0.09	0.00	0.00	0.00	0.02	0.04	0.00	0.01	0.00	0.00	0.00
T1A5	0.02	0.02	0.00	0.00	0.00	0.04	0.07	0.01	0.00	0.02	0.16	0.00	0.00	-0.02	0.00	0.00	0.00	-0.01	0.00	0.00	0.00	0.00	0.00	0.00
T1A6	0.02	0.00	0.00	0.04	0.00	0.00	0.04	0.05	0.00	0.01	0.04	0.03	0.00	-0.01	0.00	0.00	0.00	0.00	0.00	0.00	0.00	0.00	0.02	0.00
T1N1	-0.01	0.00	0.00	0.00	0.00	0.10	0.12	0.02	0.09	0.03	0.00	0.00	0.00	0.00	0.00	0.00	0.00	-0.03	0.00	0.00	-0.06	0.00	0.00	0.00
T1N2	0.00	0.00	0.05	0.00	0.00	0.00	0.00	0.00	0.00	0.12	0.00	0.17	0.00	0.00	0.00	0.00	0.00	0.00	0.00	0.00	0.00	0.00	0.00	0.00
T1N3	0.00	0.00	-0.01	0.00	0.00	0.00	0.00	0.00	0.04	0.00	0.00	-0.09	0.00	0.08	0.13	0.00	0.00	0.00	0.00	0.10	0.16	0.00	0.00	0.00
T1N4	0.00	0.00	0.00	0.07	0.00	0.00	0.00	0.01	0.03	0.02	0.00	0.10	0.00	0.03	0.00	0.10	0.00	0.04	0.00	0.03	0.00	0.00	0.00	0.00
T1N5	-0.20	0.00	0.00	0.00	0.07	0.00	0.00	0.00	0.00	0.00	0.00	0.00	0.00	0.00	0.00	0.00	0.09	0.00	0.14	0.00	0.00	0.00	0.00	0.00
T1N6	0.19	0.16	0.00	0.00	0.14	0.00	0.00	0.00	0.10	0.11	0.13	0.00	0.17	0.17	0.10	0.20	0.22	0.23	0.00	0.09	0.07	0.00	0.00	0.00
T1N7	-0.08	0.00	0.00	0.00	0.00	0.00	0.00	0.00	0.00	0.00	0.01	0.00	0.00	-0.01	0.00	0.01	0.00	-0.01	0.31	-0.12	0.00	0.00	0.00	0.00
T1N8	0.30	0.00	0.12	0.22	0.00	0.13	0.02	0.00	0.00	0.00	0.03	0.09	0.02	0.22	0.02	0.12	0.02	-0.01	0.04	0.31	0.23	0.00	0.05	0.03
T1N9	-0.13	0.00	0.00	0.00	0.00	0.00	0.00	0.00	0.00	0.00	0.00	0.00	0.00	0.00	0.06	0.00	0.00	0.00	0.13	0.00	0.17	0.00	0.02	0.00
T1N10	0.69	0.00	0.21	0.00	0.14	0.00	0.00	0.14	0.05	0.00	0.17	0.00	0.00	-0.17	0.00	0.00	0.00	-0.21	-0.30	-0.18	0.00	0.00	0.31	0.00
T1N11	-0.21	-0.11	0.00	0.00	0.00	0.00	0.00	0.00	0.00	0.00	0.00	0.00	0.00	-0.15	0.00	0.00	0.00	0.00	0.02	-0.05	0.10	0.00	0.02	0.00
T1N12	-0.31	0.00	0.00	0.00	0.00	0.00	0.00	0.00	0.00	0.00	-0.05	0.00	0.00	-0.19	0.00	0.00	0.00	0.00	-0.25	-0.18	-0.22	0.00	0.00	0.00

Note. Adjacency matrix of the T1 → T2 cross-lagged panel network in girls. Independent variables (i.e., predictors) are in columns, and dependent variables are in rows. Autoregressive edges are presented along the diagonal. A = anxiety; D = depression; N = non-suicidal self-injury
