# Supplementary material for: Longitudinal relations between non-suicidal self-injury and both depression and anxiety among senior high school adolescents: a cross-lagged panel network analysis
Source: PeerJ. 2024 Oct 7;12:e18134. doi: 10.7717/peerj.18134 (PMC11466236; doi:10.7717/peerj.18134)
Supplement: Supplemental Information 4 [file peerj-12-18134-s004.docx]

Database interpretation

“Sex” means genders; “D” means depression; “A” means anxiety;

“NSSI” means non-suicidal self injury.

“T1” means The first point in time; “T2” means The second point in time.

sex:1 =“boy”, 2=“girl”.

T1D1-T1D6: 0 = “not at all”; 1 = “A little bit ”; 2 = “Moderately ”; 3 = “Quite a bit”; 4 = “extremely”;

T1A1-T1A6:0 = “not at all”; 1 = “A little bit ”; 2 = “Moderately ”; 3 = “Quite a bit”; 4 = “extremely”;

T1NSSI1-T1NSSI12: 0 = “never”; 1 = “once in a while”; 2 = “now and then”; 3 = “frequent”; 4 = “always”;

T2D1-T2D6: 0 = “not at all”; 1 = “A little bit ”; 2 = “Moderately ”; 3 = “Quite a bit”; 4 = “extremely”;

T2A1-T2A6:0 = “not at all”; 1 = “A little bit ”; 2 = “Moderately ”; 3 = “Quite a bit”; 4 = “extremely”;

T2NSSI1-T2NSSI12: 0 = “never”; 1 = “once in a while”; 2 = “now and then”; 3 = “frequent”; 4 = “always”;

T1D = total depression scores in T1;T2D = total depression scores in T2

T1A = total anxiety scores in T1;T2A = total anxiety scores in T2

T1NSSI = total non-suicidal self injury scores in T1;

T2NSSI = total non-suicidal self injury scores in T2
